# A multiple session dataset of NIRS recordings from stroke patients controlling brain–computer interface

**DOI:** 10.1038/s41597-024-04012-6

**Published:** 2024-10-25

**Authors:** Mikhail R. Isaev, Olesya A. Mokienko, Roman Kh. Lyukmanov, Ekaterina S. Ikonnikova, Anastasiia N. Cherkasova, Natalia A. Suponeva, Michael A. Piradov, Pavel D. Bobrov

**Affiliations:** 1 Institute of Higher Nervous Activity and Neurophysiology of the Russian Academy of Sciences, Butlerova St., 5A, Moscow, 117485 Russia; 2 Research Center of Neurology, Volokolamskoe shosse, 80, Moscow, 125367 Russia

**Keywords:** Stroke, Brain-machine interface, Stroke

## Abstract

This paper presents an open dataset of over 50 hours of near infrared spectroscopy (NIRS) recordings. Fifteen stroke patients completed a total of 237 motor imagery brain–computer interface (BCI) sessions. The BCI was controlled by imagined hand movements; visual feedback was presented based on the real–time data classification results. We provide the experimental records, patient demographic profiles, clinical scores (including ARAT and Fugl–Meyer), online BCI performance, and a simple analysis of hemodynamic response. We assume that this dataset can be useful for evaluating the effectiveness of various near–infrared spectroscopy signal processing and analysis techniques in patients with cerebrovascular accidents.

## Background & Summary

Brain**–**computer interfaces (BCIs) provide a technological solution to convert data on the brain electrical or metabolic activity into control signals for an external device. BCIs can be used to provide feedback during motor imagery training (i.e. ideomotor training), which is one of the methods for motor rehabilitation after stroke^1,2^. Between 2019 and 2023, at least 11 systematic reviews were published, 8 of which included meta–analyses, demonstrating the efficacy of post**–**stroke BCI training^3–13^. It is important to note that there is a target group of patients for BCI training: those with severe paresis in the early stages after a stroke who are unable to partake in traditional physical therapy^2^. BCI technology that registers the electroencephalographic (EEG) signal accompanying the motor imagery process is the most studied for clinical application. However, EEG**–**BCI might not be practical for routine clinical use, due to high sensitivity to motion, muscle, and eye movement induced artifacts and requirement to apply conductive gels or solutions.

Near—infrared spectroscopy (NIRS) is a method of optical brain imaging that records changes in hemodynamics at a depth of up to 4 cm from the scalp. Near—infrared light (760 nm – 850 nm) is emitted through the subject’s skull, while the local changes in intensity of light absorption and scattering are recorded by a detector. The measured light intensity can be converted into estimations of cerebral total hemoglobin (HbT) and differentiated into its factions: oxygenated (HbO) and deoxygenated (HbR) hemoglobin^14^. Being much more expensive than EEG, NIRS is more convenient for practical use in a BCI circuit. It does not require electrode gel and is less sensitive to artifacts from patient movements. In addition, brain activity classification can rely on several simultaneously measured quantities, including oxy—, deoxy—, and total hemoglobin concentrations. Only a few articles have been published on the therapeutic use of NIRS—BCI after stroke^15–17^.

Due to the limited availability of NIRS technology, open access labeled NIRS datasets are highly valuable for rehabilitation BCI developers, particularly for validating machine learning and artificial intelligence algorithms for classifying brain signals. Currently, there are several available open access NIRS^18,19^ or NIRS + EEG^20,21^ datasets collected from 24–30 healthy subjects and containing 1–3 recordings from each participant. Data from stroke patients may differ due to the brain damage, potential changes in cognitive and neuropsychological functions, and older age.

To the best of our knowledge, this is the first open access dataset containing NIRS recordings from stroke patients. The dataset comprises 15 participants, 237 individual motor imagery BCI sessions utilizing three different mental tasks, over 50 hours of NIRS recordings, and 5296 trials. Each patient completed 7–24 online BCI training sessions. On average, the dataset includes 353 trials and 3.3 hours of NIRS recording data per participant.

We believe the data recorded from real stroke patients in multiple BCI sessions will be of particular interest to teams designing NIRS BCI systems for stroke rehabilitation and groups studying brain activity corresponding to motor imagery. The number of sessions is typical for a single hospitalization and allows to estimate how well cross–session transfer learning algorithms would work in real practice. The recordings from various patients could be utilized to develop and evaluate algorithms for cross–subject classification of hemodynamic activity related to motor imagery. Furthermore, the data could aid in studying the patients’ hemodynamic response to motor imagery and the response changes throughout the whole rehabilitation course.

## Methods

### Participants

This study included fifteen patients admitted to the post–stroke rehabilitation department: 9 males and 6 females, all right–handed as assessed by asking them about the utilization of the dominant hand in everyday life before stroke (no specific handedness questionnaires were used), 58.8 [49.4; 70.0] years old (median, 25% and 75% quartiles) ranging from 33 to 77 years; time since stroke onset was 7.0 [2.0; 10.0] months; all patients had one–sided cortical lesions, 8 in the left and 7 in the right hemisphere; the upper extremity Fugl–Meyer Assessment (UE–FMA) score at baseline was 47,0 [35,0; 54,0]; the Action Research Arm Test (ARAT) score at baseline was 35.0 [10.0; 44.0]. For more details refer to the Table 1. The principal objective of the pilot study that yielded the presented dataset was to evaluate the quality of NIRS-BCI control that can be achieved by stroke patients. Within a period of 1.5 years, we were able to conduct NIRS-BCI training courses for 15 patients at our clinical base. All participants were informed about the experimental procedure and gave written consent prior to the experiment. This study was conducted in accordance with the ethical standards set forth in the Helsinki Declaration. The study protocol was approved by the Local Ethics Committee of the Research Center of Neurology (approval to conduct the study and share the data, case number 7–3/22 dated August 29, 2022). The patients’ data were anonymized and depersonalized according to the local laws.

**Table 1:** Subjects characteristics and classification accuracy.

Patient ID	Sex	Age range, y.o.	Handedness	Stroke time, months	Stroke side (hemisphere)	ARAT score	FMA-UE score	Motor imagery paradigm based on the ARAT’s task	BCI training days	BCI Sessions	Each session duration, min	Total BCI exposition, min	Median classification recall	Maximal classification recall
1	m	46–50	R	≤3	right	35	58	Pinching 6 mm ball with ring finger and thumb	10	10	9.10	91	0.45	0.56
2	m	71–75	R	>6 ≤12	left	44	47	Pinching 6 mm ball with ring finger and thumb	7	7	9.10	64	0.66	0.77
3	m	56–60	R	>6 ≤12	right	35	54	Pinching 6 mm ball with ring finger and thumb	9	9	9.10	82	0.45	0.56
4	m	56–60	R	>6 ≤12	left	39	52	Pinching 6 mm ball with index finger and thumb	9	9	9.10	82	0.39	0.53
5	f	41–45	R	≤3	left	52	62	Pinching 1.5 cm ball with ring finger and thumb	10	10	9.10	91	0.49	0.65
6	m	66–70	R	>6 ≤12	left	1	11	Grasping Block 10 cm	10	17	9.10 or 13.63	214	0.62	0.71
7	m	56–60	R	≤3	right	49	52	Pinching 6 mm ball with ring finger and thumb	9	18	13.63	245	0.45	0.60
8	f	56–60	R	≤3	left	38	45	Pinching 6 mm ball with ring finger and thumb	15	18	13.63	245	0.63	0.74
9	m	76–80	R	>12	left	42	57	Pinching 6 mm ball with ring finger and thumb	8	10	13.63	136	0.50	0.56
10	f	56–60	R	>12	left	10	37	Grasping Block 7.5 cm	8	16	13.63	218	0.45	0.67
11	m	66–70	R	>3 ≤6	right	6	31	Grasping Block 10 cm	12	24	13.63	327	0.43	0.66
12	f	46–50	R	>3 ≤6	left	24	35	Pinching 1.5 cm ball with ring finger and thumb	12	24	13.63	327	0.59	0.73
13	m	66–70	R	>6 ≤12	right	46	54	Grasping stone 10*2.5*1 cm	12	23	13.63	314	0.46	0.66
14	f	66–70	R	>6 ≤12	right	4	26	Pinching 6 mm ball with ring finger and thumb	12	24	13.63	327	0.61	0.74
15	f	31–35	R	≤3	right	19	45	Gripping tube 2.25*10 cm	10	18	13.63	245	0.36	0.44

### Experimental paradigm

The physical therapist individually selected the movement type for imaging for each patient. The selected movement was the most challenging among those included in the ARAT test (refer to the Table 1). Prior to each training session, the physical therapist asked the patient to perform or attempt the target movement several times until they confirmed their readiness to mentally reproduce this movement – this is known as the priming step. If the target movement involved manipulating any ARAT subject (e.g. ball, wood block, or tube), it was provided to the patient during priming.

**Fig. 1:**
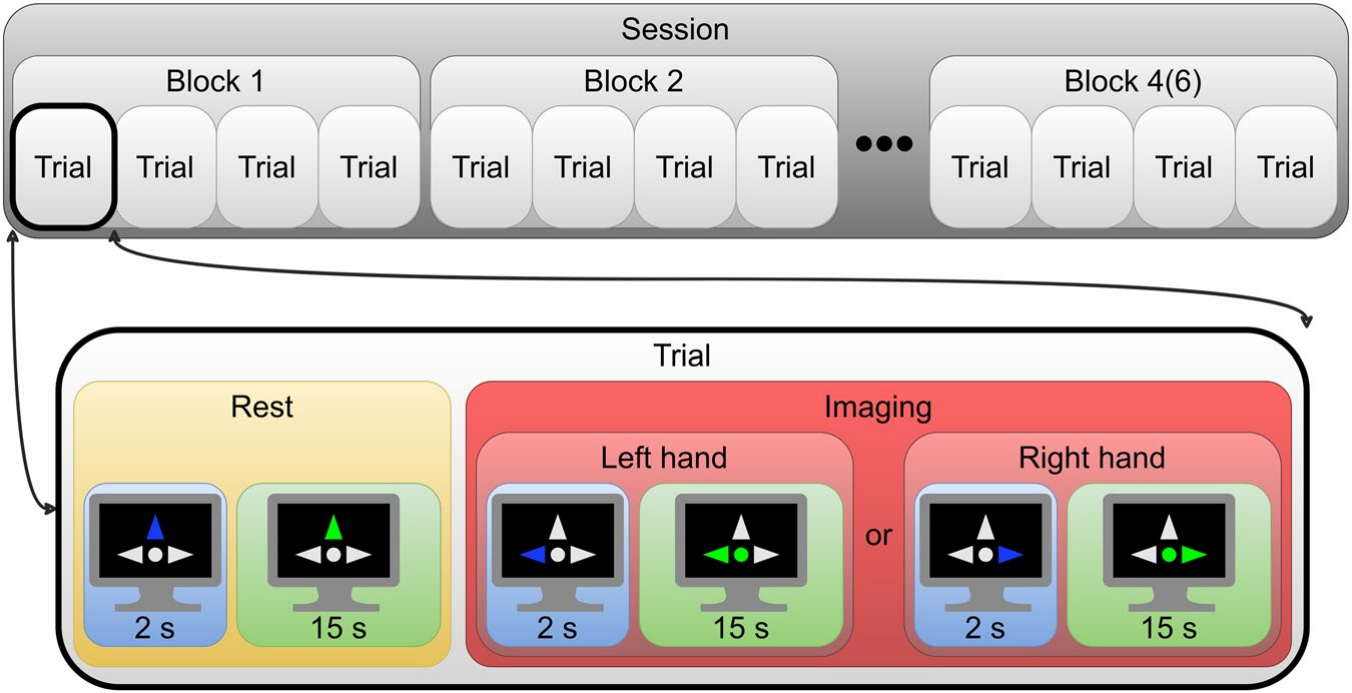
Typical session structure.

The patient, wearing a NIRS cap, sat in an armchair in front of a computer monitor with their hands resting on the armrests or the table. The screen displayed a black background with a fixation circle and three gray arrows in the center. The arrows corresponded to the tasks the patient was instructed to perform: the upper arrow indicated relaxation, while the left and right arrows corresponded to imagined movement of patient’s left and right hand, respectively. Changing the arrow color to blue served as a cue to prepare and changing the arrow color to green signaled to start performing the corresponding task. Correct classification was indicated by a green and enlarged circle, while an incorrect classification was indicated by a smaller circle. No feedback was provided when the patient had to relax or prepare. Figure 1 shows a typical session structure.

One experimental day with one patient consisted of one or two sessions. The study lasted from 7 to 15 days, with each patient participating in 7 to 24 sessions, totaling 237 sessions. A session comprised of 4 or 6 blocks and lasted 9 or 14 minutes. Each block included 4 trials: 2 right–hand movement imagining and 2 left–hand movement imagining, presented randomly. A single trial consisted of a 17–second relaxation phase followed by a 17–second movement imagery phase. During each phase, participants were given 2 seconds to prepare and 15 seconds to perform the corresponding task. Both movement imagery and relaxation were classified with overlapping epochs of 1 second and an epoch shift of 250 milliseconds. During the movement imagery, the feedback was updated based on the classification results.

### Data acquisition

**Fig. 2:**
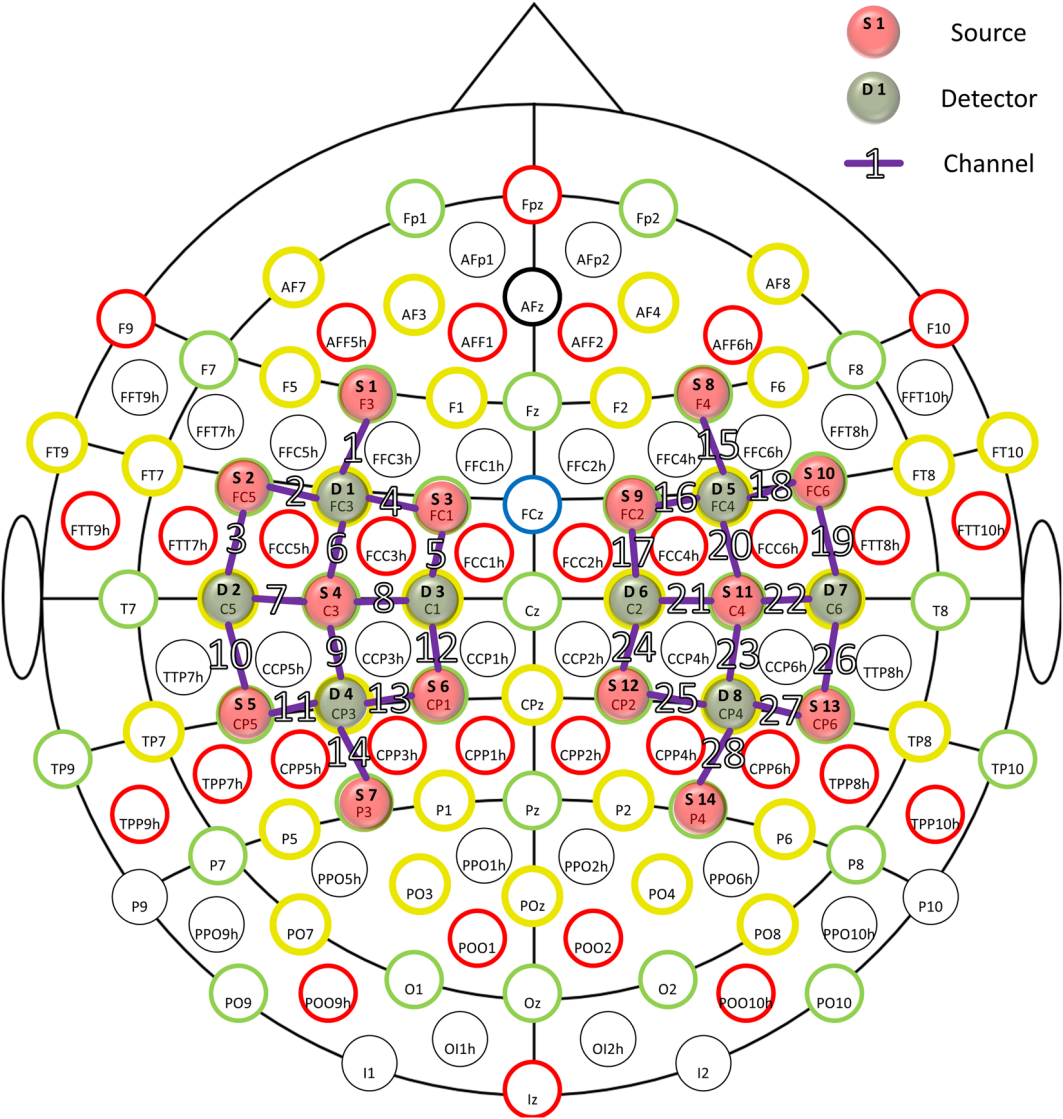
Positions of all sources, detectors, and channels. Red and green circles indicate the sources and the detectors respectively, purple lines indicate the channels.

The data were acquired using a NIRScout 16 × 8 (NIRx Medizintechnik GmbH, Berlin, Germany) in continuous wave mode of operation. Fourteen sources (LED, wavelengths of 760 and 850 nm, 5 mW per wavelength) and eight detectors (Si photodiode) were placed at a distance of about 3 cm from each other above the motor areas. Figure 2 shows the locations of all sources, detectors, and channels.

**Fig. 3:**
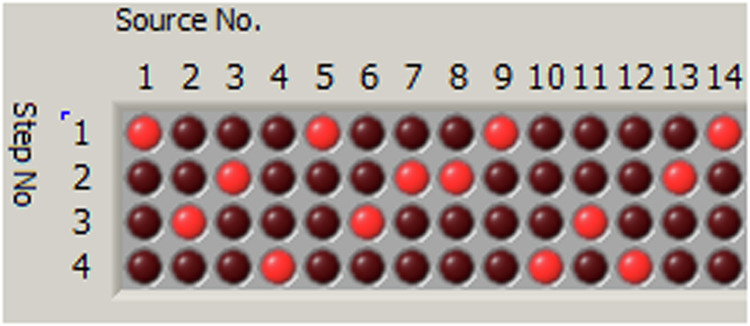
Illumination pattern. Red circles indicate light sources that were turned on at each step.

The sources were positioned at F3, FC5, FC1, C3, CP5, CP1, P3, F4, FC2, FC6, C4, CP2, CP6, P4. The detectors were positioned at FC3, C5, C1, CP3, FC4, C2, C6, CP4. A total of 28 source–detector pairs were chosen to create the NIRS channels that were recorded. Groups of light sources were turned on simultaneously giving the sampling rate 15.625 Hz (Fig. 3).

### Online data processing

NIRStar15–3 and MATLAB R2019b (MathWorks, Natick, USA) were used for data acquisition and processing. Raw data were streamed from NIRStar using Lab Streaming Layer (LSL), received using the LSL libraries for MATLAB, and processed by the BCI system implemented as a set of custom MATLAB functions. The raw NIRS data were converted to oxy– and deoxyhemoglobin relative concentrations (HbO and HbR, respectively) using the modified Beer–Lambert law (DPF = 6.2966, HbO molar extinction coefficient = 1.34956 l/(mmol*cm), HbR molar extinction coefficient = 3.56624 l/(mmol*cm) for wl = 760 nm; DPF = 5.23433, HbO molar extinction coefficient = 2.43657 l/(mmol*cm), HbR molar extinction coefficient = 1.59211 l/(mmol*cm) for wl = 850 nm; the parameters were taken from the NIRStar software). The classification was performed in two steps: first, the classifier determined whether the epoch was related to relaxation or motor imagery. Next, if motor imagery was recognized, the classifier determined which hand movement was imagined. For the first classification step, the data were band–pass filtered (2nd order Chebyshev filter) with 0.022 Hz and 0.039 Hz cutoff frequencies. These frequencies were chosen to eliminate slow, high–amplitude signal trends and minimize phase shifts on the fundamental frequency (1/34 = 0.029 Hz)^22^. Shrinkage regularization was used to avoid the adverse effects of multicollinearity. For the second classification step, the data were high–pass filtered (1st order Chebyshev filter) with 0.005 Hz cutoff frequency. Linear discriminant analysis was used to classify the data in both steps. Filtered relative concentrations (HbO and HbR) were the features. The training sample consisted of all previous blocks from the current and past sessions of the patient. The description of the classification algorithm is given in our paper^22^.

## Data Records

The dataset is freely available at the open access NeuroImaging Tools & Resources Collaboratory (NITRC) repository^23^. The data are presented in the original MATLAB format and in snirf format as recommended in the guidelines^24^. Each file name is comprised of subject ID, day number, session number, and data type specifier. The mat files contain a tabulated configuration of the channels (source–detector pairs in the 10–10 system), the raw light intensity data for each channel on both wavelengths, HbO and HbR concentrations in mmol/l, labels of mental tasks for each time point, short descriptions of the tasks, the time vector, sampling rate, confusion matrix resulting from online classification, and classification accuracy metric (as summarized in Table 2).

**Table 2:** List of the variables stored in mat data files.

Data type specifier	Variable name	Description
raw, concentrations	Channels	Source and detector labels in 10-10 system for each channel
raw, concentrations	Frequency	Sampling rate in Hz
raw, concentrations	Tasks	Short description of each task
raw, concentrations	Tasks_labels	Task number for each time frame. Frames labeled with 0 were not used for classification
raw, concentrations	Time	Time vector specifying time in seconds for each frame
concentrations	HbO	Relative concentrations of HbO for each channel and time frame
concentrations	HbR	Relative concentrations of HbR for each channel and time frame
concentrations	Concentrations	Units of HbO and HbR concentrations
raw	Wavelength1	Raw 760 nm light intensity data streamed from NIRStar
raw	Wavelength2	Raw 850 nm light intensity data streamed from NIRStar
cls	Confusion_matrix	3 × 3 matrix with columns representing the tasks given and rows representing the BCI classifier answers. Each element is a number of times the classifier made the corresponding answer while the corresponding task was cued
cls	Recall	Online classification performance metric

## Technical Validation

**Fig. 4:**
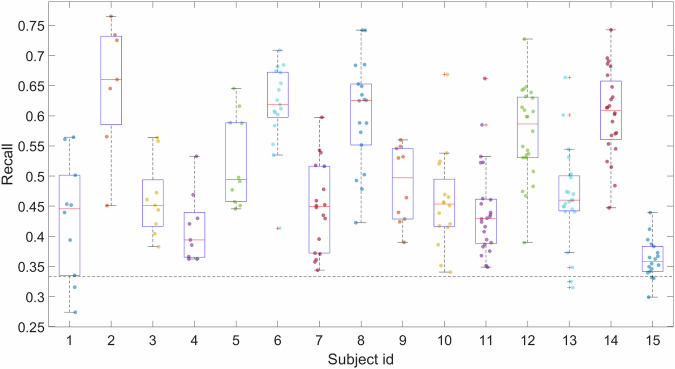
Online classification performance of all subjects. Dots indicate sessions, red lines indicate medians for each subject, boxes indicate 25% and 75% quartiles, dash line indicates random chance level.

Recall $P=\left(\mathop{\sum }\limits_{i=1}^{3}\left({c}_{{ii}}/\mathop{\sum }\limits_{j=1}^{3}{c}_{{ji}}\right)\right)/3$, where *c*_*ij*_ are the elements of the confusion matrix, was chosen as a metric for online classification performance. The grand median recall for online classification of all subjects was 46.0 [44.7; 60.3]%. All subjects exceeded the random classification level. Classification performance varied significantly between patients and between sessions for most subjects (Fig. 4). The median within–subject range of session recall was 29.5 [18.6; 31.8]%.

**Fig. 5:**
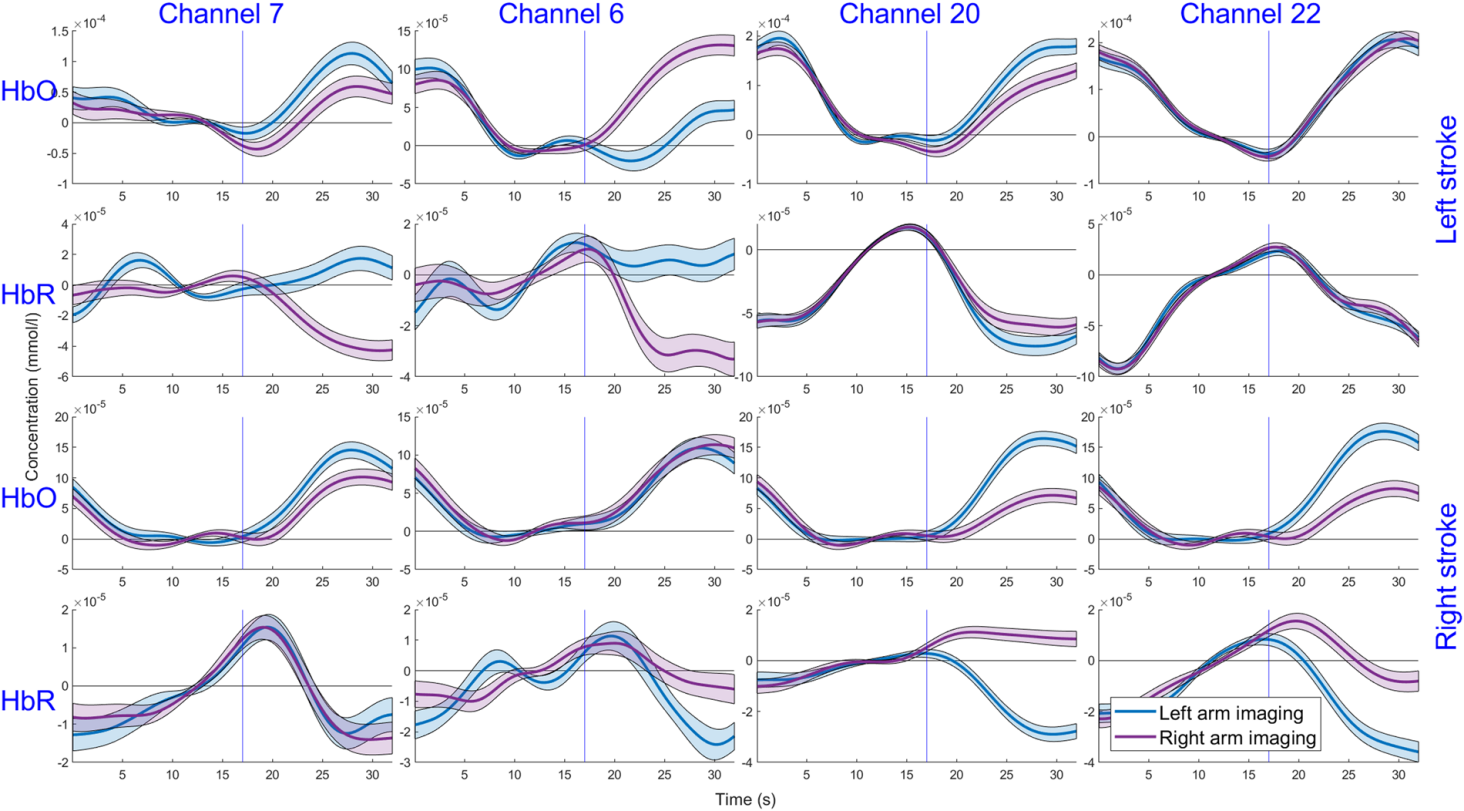
Hemodynamic responses during rest and imaging states at channels 6 and 7 from the left hemisphere and symmetric channels 20 and 22 from the right hemisphere for patients with left– and right–side strokes. The blue and red lines indicate left– and right–hand imaging. Semi–transparent areas show standard error and blue vertical line indicates movement imaging start.

To plot the responses during left or right imaging, the data were zero phase band–pass filtered (4nd order Chebyshev filter) with 0.005 Hz and 0.09 Hz cutoff frequencies, and each response was baselined by subtracting the average value of the last 10 seconds before the task began. Averaging HbO and HbR responses separately for patients with left– and right–lesioned hemisphere shows that the response is present to the imaging of both hands in both hemispheres (Fig. 5). At the same time, the response is similar for the imaging of both the paretic and intact hand in the intact hemisphere. However, there is an asymmetry of the response in the affected hemisphere, with a greater response to the imaging of the paretic hand. We suppose this asymmetry of hemodynamic response is due to a greater interhemispheric inhibitory drive from the intact hemisphere to the lesioned one^25,26^.

## Usage Notes

This dataset is licensed under the Creative Commons Attribution (CC-BY).

One of the main disadvantages of the dataset is its unbalanced design (4 or 6 blocks per session and a different number of sessions for patients). Unfortunately, this is how real rehabilitation procedures look like: for various reasons patients stop participating in the experiment. If one wants to analyze balanced data, one can simply discard the extra blocks and sessions, the experiment design allows this to be done.

## Data Availability

The MATLAB code used to create figures (Figs. 4, 5) is freely available at the open access repository^23^.
