# Correction: A multiple session dataset of NIRS recordings from stroke patients controlling brain–computer interface

**DOI:** 10.1038/s41597-025-05466-y

**Published:** 2025-07-02

**Authors:** Mikhail R. Isaev, Olesya A. Mokienko, Roman Kh. Lyukmanov, Ekaterina S. Ikonnikova, Anastasiia N. Cherkasova, Natalia A. Suponeva, Michael A. Piradov, Pavel D. Bobrov

**Affiliations:** 1 Institute of Higher Nervous Activity and Neurophysiology of the Russian Academy of Sciences, Butlerova St., 5A, Moscow, 117485 Russia; 2 Research Center of Neurology, Volokolamskoe shosse, 80, Moscow, 125367 Russia

Correction to: *Scientific Data* 10.1038/s41597-024-04012-6, published online 25 October 2024

In this article there was an error in the Local Ethics Committee protocol number. The sentence currently reads, ‘The study protocol was approved by the Local Ethics Committee of the Research Center of Neurology (approval to conduct the study and share the data, case number 5–4/22 dated June 1, 2022)’ and should read ‘The study protocol was approved by the Local Ethics Committee of the Research Center of Neurology (approval to conduct the study and share the data, case number 7–3/22 dated August 29, 2022)’. The original article has been corrected.

